# N-of-1 Trials, Their Reporting Guidelines, and the Advancement of Open Science Principles

**DOI:** 10.1162/99608f92.a65a257a

**Published:** 2022-09-08

**Authors:** Antony Porcino, Sunita Vohra

**Affiliations:** 1 EPICC, Supportive Cancer Care, BC Cancer Agency, Suite 200, 601 West Broadway, Vancouver, BC. V5Z 4C2. Canada.; 2 Dept of Pediatrics and Psychiatry, Faculty of Medicine & Dentistry, University of Alberta, 4-584 Edmonton Clinic Health Academy, 11405-87 Ave NW, Edmonton, AB, T6G 1C9. Canada.

**Keywords:** N-of-1 trials, single case experimental designs, reporting guidelines, open science, scholarly communication, reproducible research

## Abstract

N-of-1 trials are multiple crossover trials done over time within a single person; they can also be done with a series of individuals. Their focus on the individual as the unit of analysis maintains statistical power while accommodating greater differences between patients than most standard clinical trials. This makes them particularly useful in rare diseases, while also being applicable across many health conditions and populations. Best practices recommend the use of reporting guidelines to publish research in a standardized and transparent fashion. N-of-1 trials have the SPIRIT extension for N-of-1 protocols (SPENT) and the CONSORT extension for N-of-1 trials (CENT).

Open science is a recent movement focused on making scientific knowledge fully available to anyone, increasing collaboration, and sharing of scientific efforts. Open science goals increase research transparency, rigor, and reproducibility, and reduce research waste. Many organizations and articles focus on specific aspects of open science, for example, open access publishing. Throughout the trajectory of research (idea, development, running a trial, analysis, publication, dissemination, knowledge translation/reflection), many open science ideals are addressed by the individual-focused nature of N-of-1 trials, including issues such as patient perspectives in research development, personalization, and publications, enhanced equity from the broader inclusion criteria possible, and easier remote trials options. However, N-of-1 trials also help us understand areas of caution, such as monitoring of post hoc analyses and the nuances of confidentiality for rare diseases in open data sharing. The N-of-1 reporting guidelines encourage rigor and transparency of N-of-1 considerations for key aspects of the research trajectory.

## 1. Introduction

Like most clinical research, the design and use of N-of-1 clinical trials predates the current digital research environment. N-of-1 trials are multiple crossover trials in individuals (done over time within a single person), distinguishing them from within-person trials, which involve a trial on two body parts (e.g., opposite thighs) of the same person; they can also be done as a series of trials involving many individuals. To explore the design further, please refer to (Epstein & Dallery, 2022) article in this issue, and (Mirza et al., 2017) article on the history and development of N-of-1 trials. As per this issue’s introduction, scientific language is not always patient-friendly or plain language. In support of that concern, we recommend using language appropriate to the audience, such as ‘personalized trials’ or similar language for patients and clinical health care providers, and N-of-1 trials as the formal research nomenclature for the trial design as used herein.

N-of-1 trials are very robust to high heterogeneity of effect between patients, non-ideal conditions, and differing co-morbidities because the unit of analysis is the individual (Zucker et al., 1997). This allows the trials to accommodate a broader range of individuals and maintain power. N-of-1 trials are particularly useful in rare diseases and pediatrics, but they are also relevant across a wide range of chronic health conditions, with multiple published trials in, for example, osteoarthritis, insomnia, neuropathic pain, and hypertension (n.d.-a)(Punja et al., 2016)(n.d.-b). Due to their rigor and relevance, N-of-1 trials have been included in the highest level of evidence from the Oxford Centre of Evidence-based Medicine (n.d.-c).

Two critical recent advances in research include the open science movement and the use of reporting guidelines to increase transparency, accountability, and reproducibility. Unique features of the N-of-1 trial, including its patient-centered approach and possible confidentiality issues, warrant further consideration from the open science and publishing guidelines perspective.

Open science efforts were underway in the late 20th century, documented by the 1999 UNESCO/ICSU Declaration on Science and the Use of Scientific Knowledge and the Science Agenda, and followed by the 2002 Budapest Open Access Initiative, the 2003 Bethesda Statement on Open Access Publishing, and the 2003 Berlin Declaration on Open Access to Knowledge in the Sciences and Humanities. A useful, current guide is the *Draft Text of the UNESCO Recommendation on Open Science* from their Intergovernmental Meeting of Experts in May 2021 (n.d.-d)

The purpose of open science is to make scientific knowledge fully available to and ‘reusable’ by anyone, increasing the collaboration and sharing of scientific efforts and of standards in research. Application of these ideals and standards should increase transparency, rigor, and reproducibility, and reduce research waste (Bartling & Friesike, 2014)(Eisfeld-Reschke et al., 2014)(Fecher & Friesike, 2014)(n.d.-e). Four key domains of open science address equity in open access to scientific knowledge:

- scientific publications (many forms),
- research data,
- software and source code, and
- hardware.

The research contents within these domains are, optimally, released under a license that permits reuse, repurpose, adaptation, and redistribution by others with appropriate attribution. These domains are broad, and there are now many organizations developing structures, databases, and recommendations within each to help researchers implement open science approaches and ideals. Open access publishing is a well-established example. Together, these domains provide for continuity of open science practices for all stages of a research project.

(n.d.-f) developed the “Wheel of Open Science Practices” to examine how research trials can address open science from conception to completion. Individual items in the Wheel are not an exhaustive list, but rather a detailed example set of open science practices relevant to a complete research workflow. The workflow domains, *Discovery* ➔ *Analysis* ➔ *Writing* ➔ *Publication* ➔ *Outreach* ➔ *Assessment*, were developed with a broad range of research stakeholders at workshops throughout 2016. These categories facilitate consideration of specific issues for N-of-1 trials in the context of open science.

An important parallel development to open science is the emergence of reporting guidelines—a minimum set of items that should be included in a published article. Reporting guidelines promote transparency, rigor, and reproducibility, while reducing research waste (duplication of work, hidden results, and loss of unpublished results) by focusing on reporting standards (Altman & Moher, 2014), including the registration of trials and the publication of protocols. One of the first key reporting guidelines was the CONSORT Statement (Consolidated Standard of Reporting Trials) 1996, updated in 2010 (Schulz et al., 2010), providing an internationally developed and recognized reporting standard for trials as well as a rigorous method for developing further guidelines (Moher et al., 2010). There is now an online repository, the Equator Network, with 483 reporting guidelines (as of November 21, 2021). Some are fundamental guidelines such as CONSORT, SPIRIT (Standard Protocol Items: Recommendations for Interventional Trials), and PRISMA (Preferred Reporting Items for Systematic Reviews and Meta-Analyses), and many are *extensions* to those fundamental guidelines. Extensions provide specific guidance relevant to a particular situation, such as medical specialty (e.g., oncology, pediatrics) or aspects of research such as methodological subtypes and issues (e.g., crossover trials, adverse effects, interventions and intervention type, analyses, and use of patient reported outcomes). N-of-1 trials have two linked extensions: the SPENT (SPIRIT Extension for N-of-1 Trials, for trial protocols) (Porcino et al., 2020) and CENT (CONSORT Extension for N-of-1 Trials, for completed trials) (Vohra et al., 2015) guidelines, effectively covering the typical life of an N-of-1 trial from conception to publication.

## 2. Intersections of Interest

**Figure 1.**
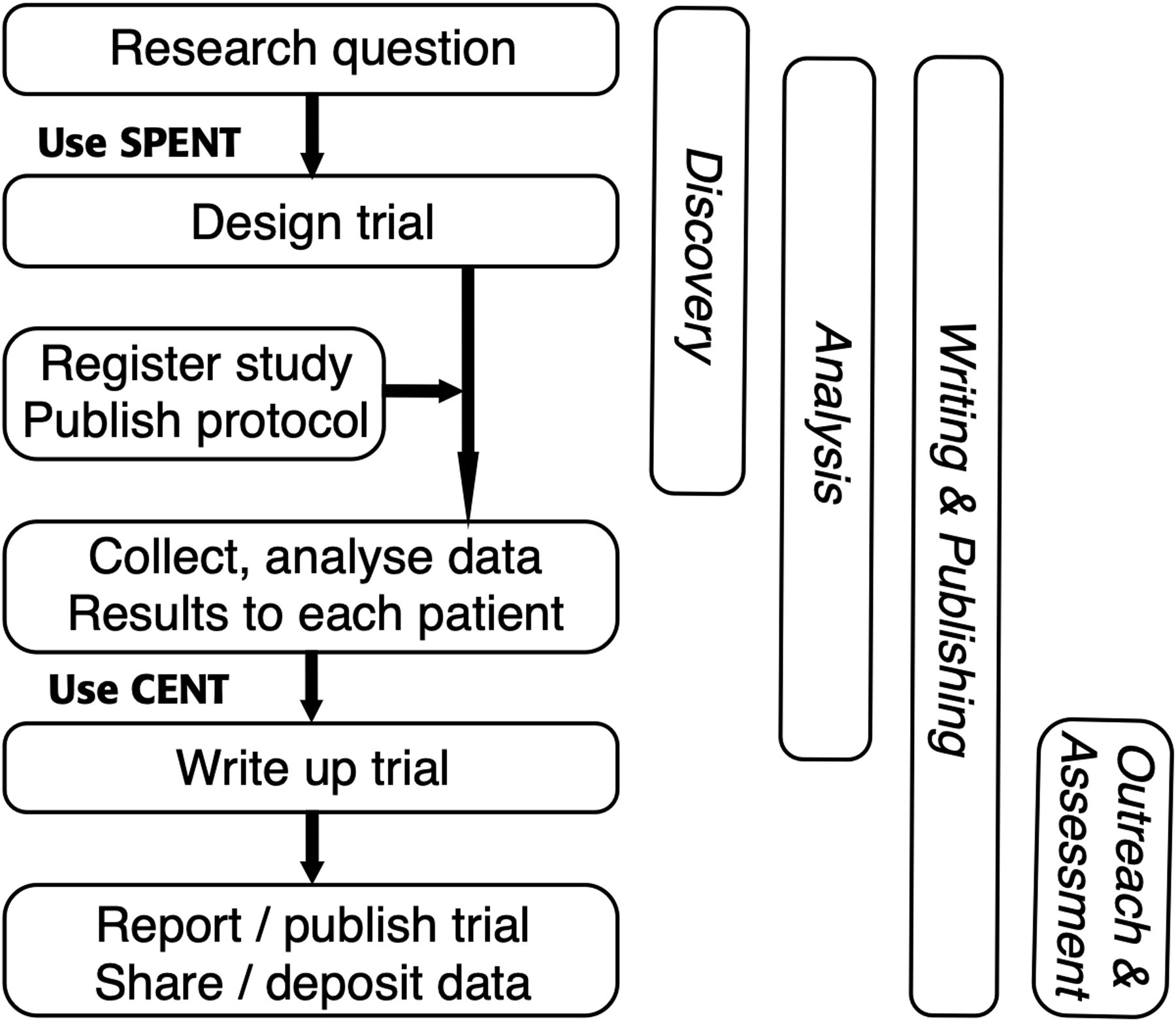
Intersection of research flow and open science processes.

Given the context of the parallel purposes of open science and reporting guidelines, it is now possible to examine specific issues within these fields for N-of-1 trials. The following schematic shows the relationship of the research and reporting flow for N-of-1 trials (as per SPENT, (Porcino et al., 2020)), and the open science workflow domains (Figure 1).

### 2.1. Discovery

Discovery addresses the beginning processes of research, including articulating the question, securing necessary funding/resources, and protocol development. Because protocol reporting guidelines can be used as a framework for developing rigorous protocols, this domain is supported by recommendations of both the SPIRIT and SPENT reporting guidelines. Reviewing both the protocol and reporting guidelines at this stage helps ensure that whatever is developed is built with the end results and dissemination needs in mind. Additionally, publication of the protocol is an important aspect of open science, sharing knowledge of the work being developed and providing the baseline for review of results in the final ‘assessment’ domain, lessening research waste.

The patient-centered nature of N-of-1 trials addresses the key issues of equity and openness in research through the inclusion of patients/caregivers in the development of a trial protocol, and as co-researchers throughout the trial. Early sharing of ideas, trial development, and protocol publications can be enhanced by sharing through multiple channels (e.g., blogs, interactive social media, online discussions, as well as through traditional academic channels), allowing better awareness, knowledge, and participation by more persons interested in the work (researchers, health care providers, patients, and caregivers).

N-of-1 trials can be built around patient-specific issues, and should specifically include patient priorities and goals, addressing questions relevant to the patients involved (Porcino et al., 2020). Thus, patients should be included in discussions on treatment options and preferences, treatment delivery, and outcome measures, if the results are to be seen as relevant by them (Kravitz et al., 2014). As such, in some trials, the same patients could serve both as research co-developers as well as participants. The trial’s robustness to high heterogeneity of effects allows inclusion of a broader range of patients (Zucker et al., 1997), facilitating equity in research. Due to the broader inclusion criteria, results from N-of-1 trials may also be more relevant or generalizable to other patients with similar conditions (Punja et al., 2016).

Equity in turn helps address social justice (respect and equitable distribution of resources/efforts) because a smaller trial population, with a wide range of patients and health comorbidities, can still achieve relevant results: the within-person multiple crossover nature of the trial design increases power (n.d.-g). This can allow for trials of products or health care services that may be difficult to fund (e.g., off-label use, complementary medicines, pilot studies), have expensive treatments, and/or involve rare diseases for which trials may not otherwise be feasible. Because N-of-1 trials are smaller, we have found them to be less expensive to undertake compared to a similarly-powered parallel-group trial. Clinical trials are only as representative as the populations studied; by addressing questions that are relevant to patients from diverse ethnic and socioeconomic backgrounds, as well as health conditions, N-of-1 trials could enable and facilitate the development of evidence that is relevant to more diverse peoples. Acknowledging the patient-researchers in the contributor roles section (if not co-investigators/authors), if they so desire, is also important to the consideration of social justice, equity, and the relevance of the work to patients from their perspective.

There are ways both SPENT and CENT support open science goals, starting with this early phase of research. Both recommend clear titles using N-of-1 in the title to make finding and identifying these trials easier (Item 1a in both), as well as trial registration (SPENT Item 2a & b, CENT 23). Design recommendations include careful articulation of the N-of-1 period details, sequences, and randomization processes, including run-in and washout details for the medications between the periods (treatment cycles) (SPENT: Items 11a–d, 14, 15, 16a–c; CENT: Items 5–11), together increasing understanding of the trial design and reproducibility. SPENT recommends documenting the statistical analysis plan, including subgroup or adjusted analyses (Items 20a–c), to limit post hoc analyses and provide a clear source of verification when reviewing published results (CENT Items 12a–c). SPENT also encourages planning for sharing of results with the research participants as well as broader dissemination of results and plans for the sharing of data.

### 2.2. Analysis

Analysis represents all the steps involved in doing the research project. An important aspect of open science is the constant recording of steps, inputs, and data. The production and reporting of data—the data sets themselves, related digital research objects (such as plans and workflows), and the management and stewardship of such data—are addressed with the FAIR Guiding Principles: findability, accessibility, interoperability, and reusability (Wilkinson et al., 2016). These should be applied to both the meta-data and tangible data to assist discovery and reuse; these principles of FAIRness can also be applied to other aspects of open science (e.g., (Battaglia et al., 2019)).

Open science recommends open sharing of data and results throughout the data collection period and into the writing, publishing, and post research assessment/knowledge translation periods. The Open Data Institute’s *Data Ethics Canvas* notes that data sharing must be carefully planned, and specifically can have negative effects on people (n.d.-h). While not unique to N-of-1 trials, observing patient confidentiality and limiting identifying information can become more complex for N-of-1 trials, particularly when dealing with rare diseases or other focused populations because participants could potentially be identified from information such as the condition under study, other demographic information, study setting(s), and/or comorbidities. In some situations, even in the best scenarios, it may be difficult to fully protect confidentiality. Both SPENT (Item 27) and CENT (introduction to the checklist) contain specific recommendations and examples addressing this issue. Consideration of confidentiality must begin in planning, with the possible confidentiality issues carefully balancing open science’s drive for equal and open sharing of all data and trial information. In some trials, a stated willingness to share data rather than full publication may be more prudent. When patient confidentiality and anonymity may be compromised through data sharing, it is even more important to seek and obtain patient consent prior to making their data available to others. Should potential ethical issues be recognized during planning for data sharing, the concerns and mitigating steps should be documented in the consent, and your local ethical review board consulted as needed. The FAIR guidelines support this also, in recognizing that different levels of data may require different degrees of openness and must be planned for in advance (Wilkinson et al., 2016).

Reporting guidelines are silent on the open publishing of data either during process or as part of reporting. However, if reporting guidelines like SPIRIT and SPENT are used, specific plans for recording and storing all aspects of the trial, as well as decision points and processes (e.g., stopping rules: SPENT Items 11b, 21b) during the research, should be planned ahead as part of the protocol. Similarly, if CONSORT and CENT were considered during development of the project, all aspects of data capture and reporting will have been planned in advance for meeting reporting expectations. Use of the FAIR Guiding Principles (Wilkinson et al., 2016) would complement the reporting guidelines, by their necessary preplanning for data capture with FAIR-guided data sheets, dictionaries, consideration of metadata, and publication/storage of the research output. Consideration of potential ethical concerns, such as the possibility of participant identification, can therefore be planned for early in the research process. Thus, applying the reporting and FAIR guideline recommendations from the start of the research will help with rigor throughout the research process, as well as facilitate open science best practices.

### 2.3. Writing and Publishing

These two domains represent a continuum. Both protocols and results should be written and published, using the SPENT and CENT guidelines and FAIR principles as explained above. Open science encourages publication of all results and data, even when results are not significant, in an effort to reduce research waste and unnecessary duplication, increase understanding of the topic, and reduce publication bias (selective publication of positive results). Publication of a trial protocol, using a reporting guideline such as SPENT, makes it easier to identify unreported research results.

As described above, open science would suggest posting descriptions of ideas early in the development process, along with early data. Because of the nature and individual focus of N-of-1 trials, early phase descriptions may help identify additional potential partnerships and participants from a much broader population base, particularly if using social media channels to share the initiative. Additionally, being based on individuals, additional trial participants could more easily be added remotely, increasing the trial size without significant additional research costs (Nikles et al., 2006).

N-of-1 trials are also used in clinical care to address specific issues in patient treatment, such as reducing ineffective polypharmacy, or situations where the best choice is not clear, perhaps due to comorbidities, off-label pharmaceutical use, or lack of relevant available evidence (Guyatt et al., 1988). Aside from the occasional published case report, much of what is learned from these clinical care trials is lost. Open science principles would suggest that the protocols developed and the data gathered have scientific value and sharing should be encouraged and supported. Having a repository of N-of-1 trials, open to N-of-1 trials done as research or as part of clinical care (for further elucidation of the distinction in N-of-1 trials work, please see (Punja et al., 2014)), indexed by condition and intervention, would overcome this loss and would facilitate open science’s goals by expediting the growth of knowledge with each patient’s trial results. Using the FAIR Guiding Principles to guide the development of N-of-1 trials meta-data identifiers and vocabulary would be a good starting point, and would help with information standardization in clinical trial registries.

### 2.4. Outreach and Assessment

Outreach looks beyond just publishing though the usual routes (e.g., journals, reports), and into the many aspects of knowledge sharing, translation, and transfer. This includes ideas such as ensuring that the patient populations involved can learn about the results of the work through patient-relevant and accessible channels (e.g., social media) but also that the data are presented in ways understandable by the patients and appropriate levels of language are used for sharing. For example, visual representation of the data has been shown to increase comprehension and learning (Bobek & Tversky, 2016)(n.d.-i) and are thus recommended for N-of-1 trials. Because the trials are focused on addressing issues in an individual, reporting back to the patients involved is expected (Porcino et al., 2020)(Vohra et al., 2015).

Assessment, the reflection on the value and processes of the research, publication, and outreach, is a worthwhile endeavor. It includes work to assess the quality and learning from research, such as systematic reviews and meta-analyses, and work to avoid research waste. N-of-1 trials can, and should, be included in systematic review and meta-analyses to better inform estimates of treatment effect (n.d.-j) as well as open science efforts to use all the data and knowledge available to better inform care.

## 3. Conclusion

The goals of open science are to conceptually shift the production, documentation, and sharing of data in a way that supports an open and respectful advancement of science while reducing research waste and reporting bias. Key processes involve ongoing and open sharing of ideas, identities, processes, and data. Reporting guidelines are one well-established tool that, by encouraging standardized and clear reporting at the beginning and end of the research process, align well with the goals of open science. N-of-1 trials are only one type of trial, but their emphasis on personalizing research, reducing research waste, and increasing research equity and justice provide an easy route for conceptualizing and applying some open science concepts. With modern technological developments including calls for increasing personalized medicine and the exploration and use of artificial intelligence (AI) in health care that could use N-of-1 trials to more rapidly increase the AI’s knowledge database, the confluence of open science initiatives and N-of-1 trials could richly add to the rapid advancement of medical knowledge and care.
